# Validation of the Fitbit Charge 5 for the Detection of Heart Rate and Electrodermal Activity

**DOI:** 10.1111/psyp.70116

**Published:** 2025-07-30

**Authors:** Katherine Ko, Genevieve McArthur, Carly Johnco

**Affiliations:** ^1^ School of Psychological Sciences, Faculty of Medicine, Health and Human Sciences Macquarie University Sydney New South Wales Australia; ^2^ Lifespan Health and Wellbeing Research Centre, Faculty of Medicine, Health and Human Sciences Macquarie University Sydney New South Wales Australia; ^3^ Australian Centre for the Advancement of Literacy, Australian Catholic University Sydney New South Wales Australia; ^4^ Dyslexia–SPELD Foundation Literacy and Clinical Services South Perth Western Australia Australia

**Keywords:** anxiety, electrodermal activity, equivital, Fitbit, heart rate, stress, validation, wearable devices

## Abstract

Wearable devices are increasingly used to evaluate psychophysiological markers of anxiety for continuous health monitoring. Consumer‐grade wearable devices, such as Fitbits, have the potential for widespread use and dissemination given their affordability and accessibility for both research and clinical settings. However, the validation of consumer‐grade devices against research‐grade devices is required. This study aimed to evaluate and compare the accuracy of the Fitbit Charge 5 against a research‐grade wearable device, the Equivital EQ02, in measuring psychophysiological parameters of anxiety, specifically heart rate (HR) and electrodermal activity (EDA). Fifty‐five undergraduate students (*M*_age_ = 19.4, SD_age_ = 1.6, 46% female) wore both Fitbit and Equivital devices whilst completing social stressor and reading tasks. Statistical analyses demonstrated significant moderate correlations between the two devices for heart rate (HR) estimates (rs = 0.45–0.58) and low to moderate correlations for electrodermal activity (EDA) estimates (rs = 0.42–0.50). Intraclass correlations were moderate for both HR (ICCs = 0.53–0.72) and EDA (ICCs = 0.46–0.64) across conditions (ps < 0.05). Furthermore, Bland–Altman analyses revealed that the Fitbit showed a pattern of underestimation of HR (ranging from 24 to 32 bpm) and overestimation of EDA (ranging from −12.92 to 10.29 μS) compared to the Equivital. These findings highlight potential reliability concerns with the Fitbit Charge 5 in measuring physiological data. While the device may have some utility in assessing HR and EDA, it is crucial to approach the interpretation of data from consumer‐grade wearable devices with caution due to potential accuracy limitations.

## Impact Statement

Our findings provide new insights into the utility and limitations of consumer‐grade wearables for monitoring psychophysiological markers of anxiety. We complement existing research by demonstrating that, while the Fitbit Charge 5 shows moderate correlations with a research‐grade device, its consistent underestimation of heart rate and overestimation of electrodermal activity indicate potential accuracy concerns. These findings emphasize the need for validation studies and cautious interpretation when using consumer‐grade devices in research and clinical applications.

Anxiety is an emotional response to internal or external stressors that can trigger an autonomic arousal response to prepare the body for “fight or flight” (Arroll and Kendrick 2018; Spielberger 1983). Increased arousal triggers physiological changes, including the release of stress hormones, which leads to elevated heart rate (HR) and the stimulation of sweat glands (i.e., increased electrodermal activity, EDA levels) (Boucsein 2012; Gordan et al. 2015). When anxiety reaches a level that impairs daily functioning, it may constitute an anxiety disorder (American Psychiatric Association 2015).

In recent years, it has become increasingly clear that anxiety can be best studied in greater depth by collecting data from daily routines. Psychophysiological markers—such as HR and EDA—can provide non‐invasive and sensitive real‐time measures of anxiety during everyday tasks (Hickey et al. 2021). Wearable systems designed for research can collect these psychophysiological measures, and there is significant interest across research, commercial, and wellness sectors in scalable, real‐time monitoring solutions to detect and track symptoms of mental illness and psychological distress (Kang and Chai 2022).

Research on psychophysiology and anxiety has typically used laboratory‐based research‐grade static systems, such as Biopac (Biopac Systems Inc., Goleta, CA) and PowerLabs (ADInstruments, Australia). These systems offer high precision and extensive functionality for controlled laboratory settings; however, they are typically limited to stationary use. In contrast, innovative research‐grade portable wearable systems, such as the Emotiv EPOC (Emotiv Systems Inc., San Francisco, CA), Hexoskin (Carré Technologies Inc.), and Empatica E4 (Empatica Inc., Boston, MA), allow for continuous, unobtrusive, ubiquitous physiological monitoring in real‐world environments, offering greater mobility and convenience (Maher et al. 2017). However, research‐grade systems have limitations for scalable use given their high cost and the complexity of data analysis and interpretation for non‐expert users.

Consumer‐grade wearable devices, like Fitbit Inc. (San Francisco, CA), Garmin Ltd. (Olathe, KS), and Apple Inc. (Cupertino, CA), have undergone research‐grade validation by comparing their data to gold‐standard laboratory equipment to determine accuracy in measuring physiological markers such as HR, sleep patterns, and physical activity levels (for reviews, see Feehan et al. 2018; Germini et al. 2022). While these devices yield acceptable accuracy, they can lack the precision of research‐grade equipment, particularly for parameters requiring high sensitivity, and typically do not allow for specific time window averaging, which can impact data analysis accuracy (De Zambotti et al. 2018; Hilden et al. 2023; Martinko et al. 2022). Additionally, these devices often provide limited contextual data, restrict access to raw data due to proprietary software, and may suffer from inconsistencies in user compliance and sensor placement, affecting data reliability (Cho et al. 2021). Still, their accessibility and low cost offer the potential to increase scalability for large‐scale studies, enhance real‐world ambulatory monitoring, and facilitate early detection and intervention, thus reducing the burden on healthcare systems (Huhn et al. 2022). Overall, wearable devices hold significant promise for enhancing health research and care, ultimately leading to improved health outcomes and personalized healthcare solutions.

When measuring physical activity, Apple and Garmin devices show high accuracy in tracking HR and other health‐related metrics during various physical activities yet yield statistically significantly lower HR estimates compared to the Polar T31 transmitter monitor (Polar Electro, Kempele, Finland) and statistically significantly higher HR estimates compared to the Parvo Medics TrueOne 2400 (Parvo Medics Inc., Sandy, UT) (Dooley et al. 2017). Fitbit devices generally demonstrate acceptable accuracy in tracking physiological markers when compared to medical‐ and research‐grade equipment, although some findings have suggested that their HR measurements can lack precision, with potential underestimations of up to 30 beats per minute (bpm), particularly during high‐intensity physical activity (Benedetto et al. 2018).

When measuring stress or anxiety, a number of studies have found acceptable measurement accuracy between Fitbit devices and gold‐standard ECG (i.e., HR) during social tasks, verbal fluency tasks, and examinations (Chalmers et al. 2021; Gagnon et al. 2022; Pakhomov et al. 2020). However, there are concerns about poorer accuracy of HR metrics at high stress levels, particularly during activities such as task preparation, oral tasks (e.g., speech performance), and arithmetic tasks, compared to measurements taken in a relaxed state (Gagnon et al. 2022). Interestingly, there is limited research assessing Fitbit's capability to measure EDA. In a single study, Ronca et al. (2023) compared the Fitbit Sense's EDA to those of the laboratory‐grade Shimmer3 EDA+ (Shimmer, Ireland) in multiple resting conditions and found a significant moderate correlation between the Fitbit Sense and Shimmer.

In summary, a handful of studies have compared HR and EDA metrics measured by Fitbit to those measured by research‐grade devices. The results suggest that Fitbit may provide accurate readings for HR under low‐level stress conditions. While a previous model, the Fitbit Sense, demonstrated moderate reliability in measuring EDA, supporting evidence remains limited. The Fitbit Charge 5, a more recent model, has yet to be validated for both HR and EDA measurement. Thus, the current study compares the accuracy of the Fitbit Charge 5's HR and EDA readings against a research‐grade reference standard wearable system (Equivital LifeMonitor EQ02) during a passive baseline period and then under ecologically valid conditions that induces state anxiety.

## 1 Methods

### 1.1 Study Design and Procedure

The reporting of this study conforms to the Strengthening the Reporting of Observational Studies in Epidemiology (STROBE) reporting guidelines (Vandenbroucke et al. 2007; Von Elm et al. 2007). The study was approved by the Macquarie University Human Research Ethics Committee (HREC). All participants provided informed consent prior to study participation. They were fitted with the EQ02 and Fitbit devices, which both continuously recorded HR and EDA throughout the session. The protocol included a baseline psychophysiological recording phase, followed by four increasingly difficult reading conditions (regular word reading, nonword reading, irregular word reading, passage reading) and then one stressful social condition (speech performance). Overall, the experiment was administered within a 30‐min period. Participants received course credit for their participation. The full study protocol was published open access on 15 February 2023 (available from https://osf.io/tu5hk/).

### 1.2 Participants

Participants were 55 undergraduate Psychology students (*M*_age_ = 19.4, SD_age_ = 1.6, range = 18–24; 85.5% female) recruited from an undergraduate research participant pool (see Table 1 for participant characteristics). Participants were screened for, and excluded if: (1) they had a history of a chronic medical condition (e.g., cardiovascular disorders, diabetes, hypertension, hypothyroidism, hyperthyroidism) or (2) they were taking any prescription medication known to affect HR or blood pressure (antidepressants, antipsychotics, or antihypertensives). Participants were instructed to refrain from smoking, consuming alcohol, or engaging in intense physical training for 24 h prior to study participation. They were also asked to refrain from eating or consuming coffee, tea, or caffeinated drinks (e.g., energy drinks) for at least 2 h before the experiment.

**TABLE 1:** Demographic characteristics of study sample.

Demographic characteristics	
Age, M (SD)	19.4 (1.6)
Sex, *N* (%)	
Females	46 (83.6)
Males	9 (16.4)
Diagnoses, *N* (%)	
Anxiety	11 (20)
Anxiety and depression	5 (9.1)
Dyslexia	1 (1.8)
Ethnicity, *N* (%)	
Comorbid	4 (7.3)
Ethnicity	
Caucasian	25 (47.3)
Asian	13 (23.6)
Middle Eastern	7 (12.7)
Indian	5 (9.1)
Mixed	3 (5.5)
Indigenous Australian or Torres Strait Islander	1 (1.8)

A priori power analysis using G*Power suggested that 35 participants would be needed for 80% power to detect an effect size of 0.4 and 0.8 for HR and EDA, respectively, based on effect sizes from Ko et al. (2024). Data were collected from 55 participants; however, technical difficulties with the Fitbit device led to substantial data loss in EDA recordings. In all cases of data loss, participants experienced significant signal dropout, with large portions of the recording period missing or unusable. As a result, complete and usable Fitbit EDA data were available for only 35 participants.

### 1.3 Measures

#### 1.3.1 Equivital EQ02 LifeMonitor

The Equivital EQ02 LifeMonitor (hereafter EQ02; Hidalgo Inc., UK; https://equivital.com/) is a research‐grade wearable system designed to continuously monitor multiple physiological parameters, including HR and EDA. The Equivital EQ02 has demonstrated strong reliability and validity for ambulatory monitoring of key physiological parameters across multiple studies. Its performance is comparable to both gold‐standard medical systems and validated wearable devices for measures such as HR, respiratory rate, skin temperature, and core temperature (Akintola et al. 2016; Liu et al. 2013). For instance, Akintola et al. (2016) reported high agreement between the EQ02 and a gold‐standard ECG system (Holter monitor) for HR and heart rate variability (HRV). Similarly, Looney et al. (2021) found that the EQ02+ accurately tracked HR across work/rest cycles, with results closely matching those of the Polar H10 chest strap, a widely validated device. These findings support the EQ02's utility as a reliable reference standard in wearable validation research.

The EQ02 has two components: the sensor chest belt and the sensor electronic module (SEM). The sensor chest belt consists of a two‐channel ECG sensor with a sampling rate of 256 Hz, which allows multichannel processing and noise reduction from the ambulatory signal. The EQ02 has a dual shoulder strap feature to ensure a comfortable fit and security during long‐term ambulation. The SEM is placed inside the sensor belt and records, stores, and transmits physiological data in real time via inbuilt Bluetooth capability to a computer for viewing and analysis.

The data collected by the SEM is downloaded and exported using Equivital Manager. For analysis, we accessed these recordings through LabChart version 8.1.30 (ADInstruments, 2023), which allowed for precise selection of relevant time windows for analysis. We defined the time windows based on the duration of each task, isolating specific intervals corresponding to task start and end times to ensure accurate data analysis. The EQ02 calculates HR at 15‐s intervals. The EDA sensor connects to the expansion port on the Equivital SEM to facilitate the recording of EDA signals, with a sampling rate of 16 Hz.

The electrode placement for the EQ02 involves placing the device on the chest so the inbuilt electrodes make contact with the skin to capture physiological signals accurately. The EQ02 add‐on EDA sensor connects to the expansion port on the Equivital SEM to enable recording of EDA signals. It was worn on the wrist with a Velcro strap and short leads terminating in snap lead connectors compatible with MLA1010 disposable electrodes. Two ECG snap electrodes were placed on the medial phalanx of the index and middle fingers (see Figure 2).

Data recorded by the EQ02 were automatically time‐ and date‐stamped (i.e., date and time in hour, minute, seconds, and milliseconds). We inserted markers into the data recordings to indicate the precise start and end times of each task. Average HR and EDA were then extracted for analysis across each task.

##### 1.3.1.1 Fitbit Charge 5

The Fitbit Charge 5 (hereafter Fitbit; San Francisco, CA; https://www.fitbit.com/) is a fitness and health tracker consisting of a number of sensors, including an optical HR monitor, a three‐axis accelerometer, red and infrared sensors for oxygen saturation (SpO_2_) monitoring, a device temperature sensor, and electrical sensors for monitoring ECG and electrodermal responses. The Fitbit was attached to the non‐dominant wrist of each participant, above the wrist bone and in close contact with the skin. Compared to other Fitbit devices, the Fitbit Charge 5 is the only model that features an EDA sensor. HR was continuously monitored using the Fitbit device, which recorded data at regular 1‐min intervals. The mean HR for each task was calculated by averaging the recorded values throughout the task. EDA was presented as the “EDA score” which indicates the mean skin conductance level (SCL). A higher EDA score signified greater psychophysiological arousal (as an index of anxiety or stress) in the participant.

Fitbit data are stored locally utilizing Bluetooth connectivity to synchronize data with the Fitbit application installed on a paired smartphone or computer. A major limitation of the Fitbit is that it is not capable of using event markers to indicate the start and end of each task. Therefore, task windows were manually identified for Fitbit recordings using timestamps from LabChart data to ensure data synchronization across devices. Fitbit HR data were collected using its built‐in optical HR sensor, which uses photoplethysmography (PPG) to estimate HR. We accessed the raw HR data using the Fitbit Web API (https://dev.fitbit.com/build/reference/web‐api/intraday/get‐heartrate‐intraday‐by‐date/), which provides intraday HR values at 1‐s or 1‐min intervals. For each task, we extracted all available HR readings within the relevant time window and computed the average HR across the full task duration. On the other hand, Fitbit EDA data were exported via the Fitbit Dashboard. When specifying a time window for EDA export, Fitbit reports four separate SCL values per window—each representing the average EDA during one of four equal quartiles of that time window. To ensure that one of these quartile values would correspond to the entire task period, we set the export window to be four times the length of each task. This way, the first quartile (Q1) output represented the average EDA across the actual task duration, while the remaining quartiles (Q2–Q4) corresponded to post‐task periods and were excluded from analysis. This method allowed us to approximate task‐specific EDA while working within the constraints of Fitbit's data aggregation format.

### 1.4 Stressor Tasks

The participants completed five stressor tasks during their lab visit, which followed a 5‐min neutral baseline condition where participants were instructed to sit quietly and relax with their eyes open. Stressor tasks included a social task and four reading‐related tasks.

#### 1.4.1 Social Task

Social tasks are well‐established methods for eliciting stress responses in experimental settings (Allen et al. 2016). In this study, a modified Trier Social Stress Test (TSST; Kirschbaum et al. 1993) was used to induce anxiety (Allen et al. 2016). Participants were asked to prepare a 5‐min speech about their ideal job and told that their speech would be videorecorded for review by a panel of experts on public speaking. Participants prepared their speech alone for 5 min, then delivered the speech in front of the research assistant and a videocamera on a tripod. Afterward, they were debriefed about the study's purpose and provided with referral information as needed. Continuous physiological recordings were taken during the preparation phase and speech phase.

#### 1.4.2 Regular Word and Nonword Reading Tasks

Reading aloud can induce mild stress especially under timed conditions (Conway et al. 2013). The Test of Word Reading Efficiency—Second Edition (TOWRE–2; Torgesen et al. 2012) measures the ability to pronounce printed words (Sight Word Efficiency) and phonemically regular non‐words (Phonemic Decoding Efficiency) accurately and fluently. The Sight Word Efficiency subtest assesses how many real words an individual can identify in 45 s, while the Phonemic Decoding Efficiency subtest measures the number of non‐words decoded in 45 s. As part of another study (Ko et al., manuscript in progress), we obtained the raw score (i.e., the number of correct words read in a specified time) and converted it to a standard score using the TOWRE–2 norms. The TOWRE–2 demonstrates high reliability (test–retest *r* = 0.89–0.93; inter‐rater *r* = 0.99) and strong validity (*r* = 0.89–0.96; Tarar et al. 2015).

#### 1.4.3 Irregular Word Reading

The Macquarie University Advanced Adult Spelling Test (MAAST; Caruana et al. 2019; www.motif.com.au) evaluates the ability to spell difficult words with irregular sound‐spelling rules. It contains 72 items, read aloud as fast as possible within 45 s. Participants were instructed to read the word list, and the total number of correctly read words was calculated.

#### 1.4.4 Passage Reading

The Reading Comprehension subtest from the York Adult Assessment Battery‐Revised (YAA‐R; Warmington et al. 2012) involves reading a 492‐word non‐fiction passage (“The History of Chocolate”) and answering 15 comprehension questions assessing knowledge, vocabulary, and inference‐making. Raw scores for reading time and accuracy are converted into scaled scores and percentiles using the normative data provided for the YAA‐R. The YAA‐R has good discriminatory power yielding 80% sensitivity and 97% specificity. For the Reading Comprehension subtest, internal consistency was *α* = 0.53 (Warmington et al. 2012).

## 2 Data Analysis

The analysis plan was pre‐registered and published as part of the experimental protocol (https://osf.io/bvdeg) with deviations outlined below. Data were analyzed using IBM Corp (2021) SPSS Statistics version 29.0 and GraphPad Prism version 10.0.2. Critical alpha was set at 0.05. Pearson product moment correlations (*r*) assessed the strength of the correlation of HR and EDA between the two devices, interpreted as: negligible (0.00–0.30), low (0.30–0.50), moderate (0.50–0.70), high (0.70–0.90), and very high (0.90–1.00) (Hinkle et al. 2003). Intraclass correlations (ICCs) were calculated using a two‐way mixed effects model to assess absolute agreement, with results classified as poor (≤ 0.50), moderate (0.50–0.75), good (0.75–0.90), and excellent (≥ 0.90) (Hinkle et al. 2003). To examine within‐participant alignment between the two devices, we conducted linear‐mixed effects models predicting Fitbit‐derived HR and EDA estimates from EQ02 estimates across tasks. Within‐subject Pearson correlations between EQ02 and Fitbit estimates were also computed for each participant to assess individual‐level agreement across tasks. Bland–Altman plots were used to provide a visual representation of device agreement and identify systematic biases, with bias (average difference) and 95% limits of agreement (Giavarina 2015).

Outliers were examined visually using scatterplots and defined as scores that were more than two standard deviations from the mean. A single outlier was detected and subsequently excluded from all analysis, resulting in a final sample size of 35 participants. Additionally, in the Regular Reading condition, one participant was omitted from the analysis due to excessive artifacts in the HR and EDA data. There were several deviations from the published protocol (https://osf.io/bvdeg). Initially, Pearson product moment correlations and Bland–Altman plots were selected as the primary statistical analysis methods. In the final analysis, we opted to expand our analysis by incorporating ICCs to enhance the comprehensiveness of our investigation. Additionally, we originally specified to exclude recordings where the data are influenced by artifacts. However, for the final analysis, we employed a more rigorous method by removing data affected by 20% outliers. This was achieved by computing the percentage of outlier data points relative to the total number of data points.

## 3 Results

### 3.1 Descriptive Statistics

Descriptive statistics for HR and EDA measurements across devices and tasks are reported in Table 2.

**TABLE 2:** Descriptive statistics for physiological measurement.

Task	HR (bpm)		EDA (μS)	
	EQ02	Fitbit	EQ02	Fitbit
	M (SD)	M (SD)	M (SD)	M (SD)
Baseline	81.98 (11.44)	77.09 (10.18)	6.32 (3.30)	8.76 (2.51)
Regular word reading	86.10 (11.27)	78.39 (13.01)	9.31 (5.04)	7.78 (2.64)
Nonword reading	84.96 (11.47)	77.79 (11.58)	9.33 (4.67)	11.36 (5.06)
Irregular word reading	86.10 (11.27)	77.49 (12.01)	9.62 (4.97)	12.93 (4.11)
Passage reading	86.13 (10.91)	76.70 (11.88)	8.63 (4.50)	10.51 (2.88)
Speech performance	88.38 (10.84)	77.76 (11.75)	10.08 (4.43)	12.32 (3.80)

### 3.2 Correlation Analyses

Pearson *r* correlations and ICCs were calculated to determine convergence between the EQ02 and Fitbit in the estimation of HR and EDA (see Table 3). Overall, we found moderate positive correlations for HR estimates (*r*s = 0.45–0.62) and low to moderate correlations for EDA estimates (*r*s = 0.36–0.50) between the two devices across conditions. ICC values for HR and EDA estimates were mostly greater than 0.5, yielding at least moderate agreement between the EQ02 and Fitbit. There was greater concordance in HR measurement between devices during the baseline period (*r* = 0.62, ICC = 0.72) compared to the stressor tasks (*r* = 0.45–0.58; ICC = 0.54–0.61). There was less differentiation for EDA, with slightly stronger correlations for EDA during the baseline period (*r* = 0.50) compared to during the stressor tasks (*r* = 0.36–0.47), as well as little difference in ICCs during baseline (*r* = 0.53) and stressor tasks (*r* = 0.46–0.64).

**TABLE 3:** Pearson *r* correlation and ICC values.

Measure	Task	Pearson *r* (95% CI)	ICC (95% CI)
Heart rate	Baseline	0.62*** (0.42–0.76)	0.72** (0.45–0.85)
	Regular word reading	0.48*** (0.24–0.66)	0.57** (0.18–0.76)
	Nonword reading	0.45*** (0.21–0.64)	0.55** (0.17–0.75)
	Irregular word reading	0.45*** (0.21–0.64)	0.53** (0.08–0.75)
	Passage reading	0.58*** (0.37–0.73)	0.61** (0.01–0.82)
	Speech performance	0.54*** (0.31–0.70)	0.54** (−0.07–0.78)
Electrodermal activity	Baseline	0.50** (0.20–0.72)	0.53** (−0.02–0.78)
	Regular word reading	0.47** (0.15–0.69)	0.53* (0.10–0.76)
	Nonword reading	0.46** (0.20–0.72)	0.64*** (0.29–0.82)
	Irregular word reading	0.42* (0.11–0.67)	0.51** (0.01–0.75)
	Passage reading	0.36* (0.03–0.63)	0.46*** (−0.02–0.72)
	Speech performance	0.43* (0.10–0.67)	0.54** (0.11–0.77)

Abbreviation: ICC, intraclass correlation coefficient.

* *p* < 0.05.

** *p* < 0.01.

*** *p* < 0.001.

### 3.3 Individual‐Level Analysis of Fitbit and EQ02 Alignment

To assess whether the Fitbit captured within‐participant physiological changes in line with the Equivital EQ02, we conducted a series of linear mixed‐effects models predicting Fitbit‐derived EDA and HR values from EQ02 values across tasks, with participant included as a random effect to account for repeated measures. Results indicated that Fitbit HR closely mirrored EQ02 HR fluctuations at the group level (*B* = 0.91, SE = 0.04, *p* < 0.001), suggesting strong correspondence in HR reactivity across tasks. Similarly, Fitbit EDA showed a significant positive association with EQ02 EDA (*B* = 0.72, SE = 0.09, *p* < 0.001), indicating that Fitbit tracked within‐subject changes in EDA across conditions.

However, to assess device agreement at the individual level, we computed within‐subject Pearson correlations between EQ02 and Fitbit values across tasks for each participant. None of the individual correlations reached statistical significance (all *p*s > 0.05), suggesting that despite capturing general within‐subject trends at the group level, the Fitbit may not reliably reflect task‐related physiological changes at the level of individual participants.

### 3.4 Bland–Altman Analyses

**FIGURE 1:**
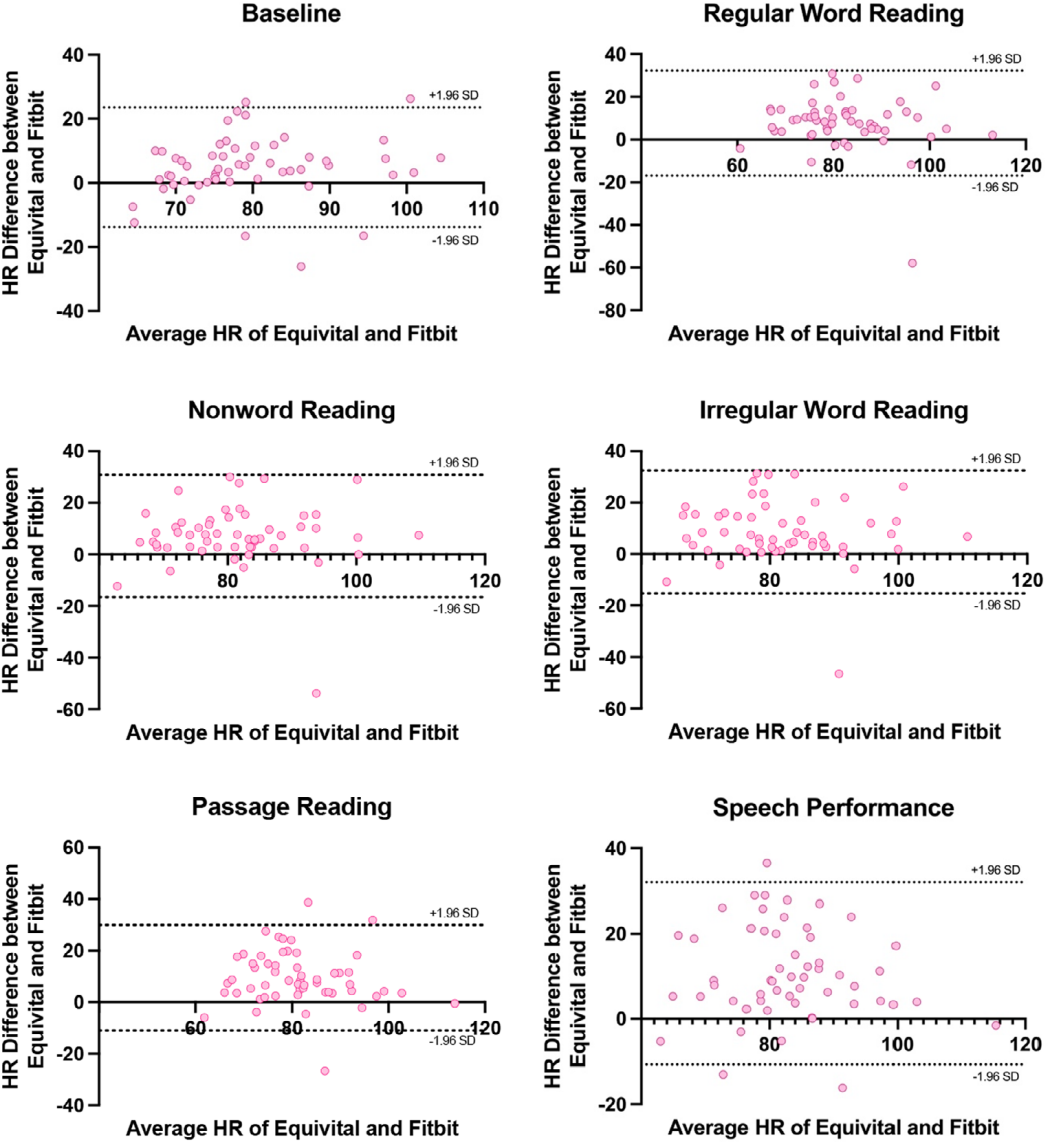
Bland–Altman plots on the agreement between the EQ02 and Fitbit for HR estimates. Each dot represents paired (Fitbit–EQ02) HR values derived from all participants. The 95% limits of agreement are shown as two dotted lines.

The Bland–Altman plots provide a visual representation of the systematic differences between the EQ02 and Fitbit. Figure 1 presents Bland–Altman plots on the agreement between the EQ02 and Fitbit for HR estimates, with results summarized in Table 4. The findings indicate a consistent trend of underestimation by the Fitbit in comparison to the EQ02, and the magnitude of this underestimation appeared to be stable over time. The Bland–Altman analyses also revealed large biases, ranging from 4.90 to 10.62 bpm, and considerably large limits of agreement with differences between measurements of 23.59–32.26 bpm units.

**TABLE 4:** Results from Bland–Altman analyses.

Measure	Task	Bias (SD)	Limits of agreement	
			Lower	Upper
Heart rate	Baseline	4.90 (9.54)	−13.79	23.59
	Regular word reading	7.70 (12.53)	−16.86	32.26
	Nonword reading	7.17 (12.06)	−16.48	30.81
	Irregular word reading	8.61 (12.18)	−15.27	32.48
	Passage reading	9.43 (10.44)	−11.04	29.90
	Speech performance	10.62 (10.91)	−10.77	32.01
Electrodermal activity	Baseline	−2.44 (2.98)	−8.28	3.39
	Regular word reading	1.53 (4.47)	−7.24	10.29
	Nonword reading	−2.03 (4.85)	−11.54	7.48
	Irregular word reading	−3.31 (4.90)	−12.92	6.30
	Passage reading	−1.88 (4.37)	−10.45	6.69
	Speech performance	−2.25 (4.45)	−10.96	6.47

**FIGURE 2:**
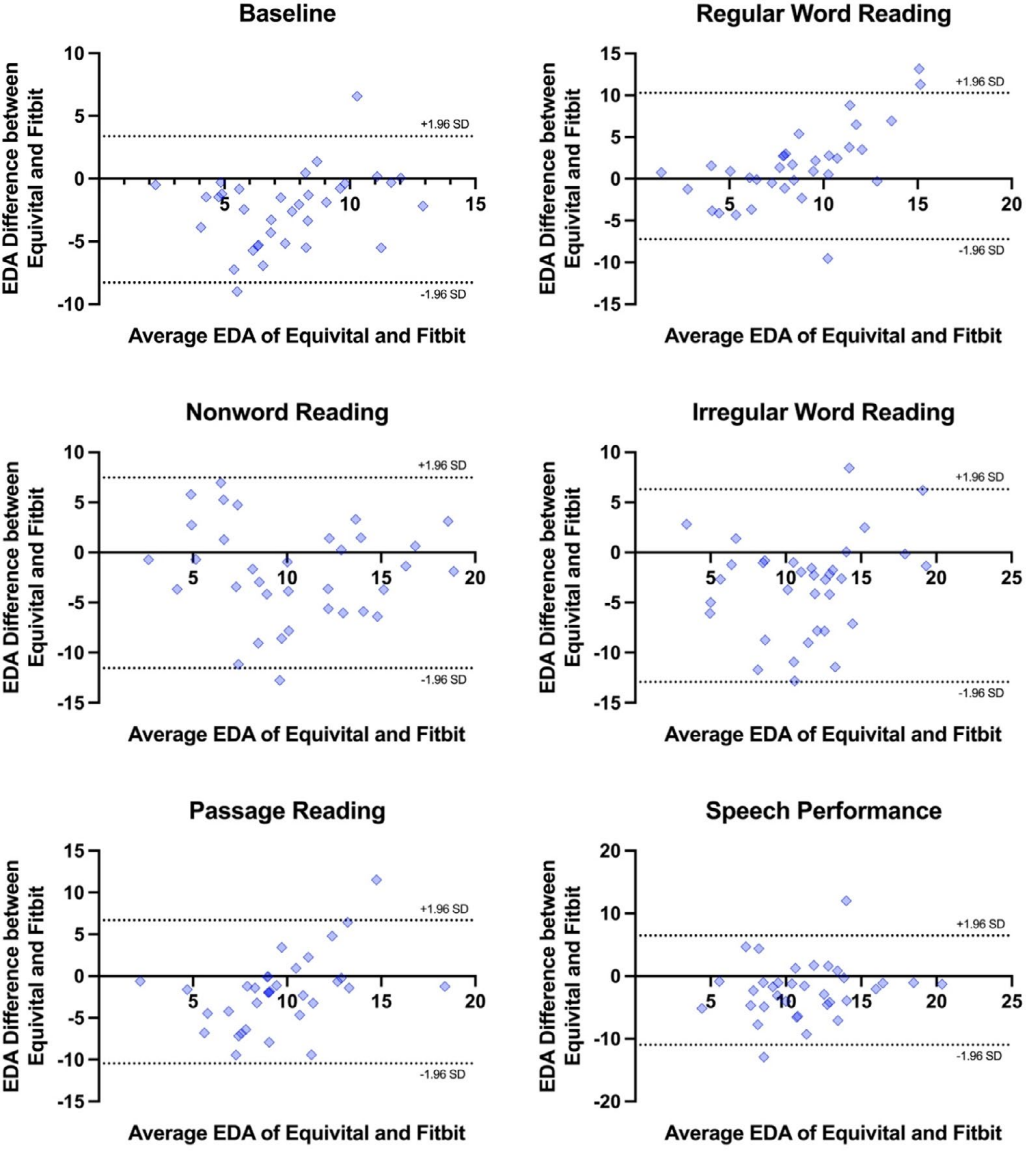
Bland–Altman plots on the agreement between the EQ02 and Fitbit for EDA estimates. Each dot represents paired (Fitbit–EQ02) GSR values derived from all participants. The 95% limits of agreement are shown as two dotted lines.

Figure 2 presents Bland–Altman plots on agreement between the EQ02 and Fitbit for EDA estimates, with results summarized in Table 4. The plots exhibit a noticeable positive slope, suggesting a consistent and systematic difference between the EDA measurements provided by the Fitbit and EQ02. Specifically, there is a greater difference between the EDA measurements at higher EDA levels compared to lower EDA levels. The Fitbit consistently yielded higher EDA measurements compared to the EQ02, with the largest bias found in the irregular word reading condition (−3.31). Biases ranged from −3.31 to 1.53 μS, with substantial limits of agreement across measures (from −12.92 to 10.29 μS), suggesting notable measurement variance.

## 4 Discussion

The current study aimed to compare the accuracy of a consumer‐grade wearable device (Fitbit Charge 5) against a research‐grade wearable system (Equivital EQ02) in measuring psychophysiological parameters, specifically HR and EDA, during a passive baseline period and then under ecologically valid conditions designed to increase state anxiety. Overall, results indicated there were at least moderate relationships between the HR and EDA measurements obtained by the two devices across a neutral baseline period, as well as during social and cognitive stressors. It is noteworthy, however, that there was also strong evidence of systematic bias as well as substantial variability and disagreement between the two devices, suggesting that they were not consistent across the measurement range. Overall, the Fitbit systematically underestimated HR and overestimated EDA compared to the EQ02 across all conditions. Further analyses showed that Fitbit measurements can broadly reflect within‐person changes in physiological arousal across different task conditions when looking at the group level. This indicates some utility in capturing general patterns of HR and EDA across tasks. However, when we examined the relationship between Fitbit and EQ02 HR data at the individual level, we found little consistency. For most participants, Fitbit HR did not closely align with EQ02 readings across tasks, suggesting that while the device may be adequate for detecting overall trends in HR, it lacks the precision needed to reliably capture moment‐to‐moment changes within individuals. This highlights an important limitation in using consumer‐grade wearables for fine‐grained psychophysiological research and suggests caution in the potential interchangeability of the two devices in measuring HR and EDA.

Upon closer examination of the pattern of the physiological data, there are evident variations in HR between the two devices across different tasks. The EQ02 consistently reported higher HR than the Fitbit. Although the Fitbit's HR measurements were aligned with EQ02's estimations, the Fitbit tended to underestimate HR. Both devices captured increased task‐related HR during stressor tasks in comparison to baseline, but the extent of change and the consistency in how these changes were measured (i.e., how reliably each device detected HR increases across different tasks) differed between the devices. Thus, the Fitbit on average showed poor to moderate agreement with the EQ02 and frequently underestimated HR by around 5–10 bpm (ranging up to 30 bpm). This aligns with previous studies (e.g., Benedetto et al. 2018; De Zambotti et al. 2018; Gagnon et al. 2022; Pakhomov et al. 2020), which indicate that Fitbit devices, including previous Charge and Versa models, showed low agreement with gold‐standard HR measurement tools. Of note, there was greater concordance in HR measurement between the two devices during baseline compared to stressor tasks, indicating that device accuracy may vary with task conditions and could be less accurate under higher stress levels (Gagnon et al. 2022). In contrast, limited research exists on Fitbit's accuracy in measuring EDA. Our findings suggest that Fitbit's EDA measurements generally align with EQ02 estimations, though with a tendency to overestimate EDA. Overall, these findings suggest that while the Fitbit demonstrates acceptable accuracy, its tendency to either underestimate or overestimate physiological parameters may diminish its utility as a tool to monitor and respond to psychophysiological increases in stress reactivity.

This study has a number of strengths, including the validation of the Fitbit in a variety of contexts, including neutral (baseline) contexts, as well as during mildly and moderately stressful environments that encompass both social and cognitive stressor tasks. Validation of a new model of a Fitbit device (Charge 5) against a research‐grade wearable system to demonstrate its effectiveness in tracking psychophysiological arousal is important. The validation of recently developed models with new features (EDA) contributes to the progress of scientific research by expanding the scope of what can be achieved with wearable technology. These developments enhance both the precision and dependability of data collection, as well as broaden access to research tools. However, there are also a number of study limitations to consider. First, there was a restricted demographic included in the sample. Participants had a limited age range, comprising mostly young adults between ages 18 and 24 years, and conclusions may not generalize to other age or demographic groups. Similarly, although around half of the sample were from non‐Caucasian ethnic backgrounds, there was insufficient power to examine ethnic differences. Second, although screening for cardiovascular conditions was conducted, we were unable to ascertain whether any participants might have experienced arrhythmias, which could potentially influence the outcomes of our study. Third, this study used a relatively small sample; thus, results warrant replication in larger samples.

Several limitations associated with the use of Fitbit devices also need to be acknowledged and addressed in future studies. Firstly, the Fitbit does not grant access to raw signals and only provides physiological measures computed by their proprietary software. As the algorithm utilized by Fitbit is protected, it is difficult to elucidate the source of the measurement discrepancies or discern whether they stem from software, firmware, or hardware components. Importantly, knowledge of these algorithms is important to ensure data integrity and will enable researchers to effectively address measurement discrepancies and biases. Plausibly, the variability in Fitbit measurements may be due to issues with the sensors or the integrated software used for metric calculation, consequently preventing the application of data processing and computation techniques that are commonplace in physiological data analysis. Moreover, artifact management is possible for the EQ02 but not the Fitbit.

Another issue associated with using wearable devices in research is the amount of data loss. During data collection, we encountered technical issues that prevented the synchronization of the Fitbit mobile application with the Fitbit device. Consequently, we were unable to consistently gather EDA data stored within the mobile application and ended up with a significantly lower number of participants with complete EDA datasets compared to those with complete HR datasets. Additionally, it is worth noting that minute‐to‐minute HR recordings can be retrieved via the Fitbit Web API, but minute‐to‐minute EDA data can only be accessed through the mobile application. While research‐grade wearable sensors offer greater accuracy and a wider range of precision measurements, consumer wearables have the advantage of being more affordable, accessible, and scalable. Exploring the range of consumer‐grade wearables and evaluating their efficacy against research‐grade devices could yield valuable insights into the potential limitations of various devices in accurately capturing psychophysiological responses to anxiety and stress. This comparative analysis could provide important information for device selection. Furthermore, longitudinal research and large‐scale validation studies are warranted to assess the long‐term reliability and validity of Fitbit devices for continuous monitoring of physiological measures. Understanding how these devices perform over extended periods of use is critical for evaluating their suitability in research and clinical applications, as well as the potential impact of user behavior, environmental factors, and device wear and tear on data accuracy and reliability over time.

With the growing accessibility and increased interest in the use of wearable technology for mental health monitoring, it is crucial to establish their accuracy for tracking psychophysiological data. Compared to laboratory‐ or research‐grade wearable devices, consumer wearable devices are prone to issues with accurate measurement of physiological parameters, such as HR and EDA. The current findings suggest that the Fitbit Charge 5 exhibits at least a moderate level of accuracy for measurement of HR and EDA compared to research‐grade devices, although it tends to under‐ and over‐estimate HR and EDA, respectively. As such, there may be circumstances where the Fitbit Charge 5 may offer utility for assessment of HR and EDA; however, it is important to maintain a cautious approach towards interpreting data derived from consumer‐grade wearable devices given potential issues with data accuracy.

## Author Contributions

**Katherine Ko:** conceptualization, methodology, investigation, formal analysis, funding acquisition, writing – original draft, writing – review and editing. **Genevieve McArthur:** conceptualization, methodology, investigation, writing – review and editing, supervision. **Carly Johnco:** supervision, conceptualization, methodology, investigation, writing – review and editing.

## Conflicts of Interest

The authors declare no conflicts of interest.

## Data Availability

The data that support the findings of this study are openly available in A Validation of Physiological Measures of Anxiety in Adults at https://osf.io/tu5hk/?view_only=e66630f3d0ef47c99881398dcda4e1e6.
